# The Association Between Habitual Sleep Duration and Mortality According to Sex and Age: The Japan Public Health Center-based Prospective Study

**DOI:** 10.2188/jea.JE20190210

**Published:** 2021-02-05

**Authors:** Thomas Svensson, Manami Inoue, Eiko Saito, Norie Sawada, Hiroyasu Iso, Tetsuya Mizoue, Atsushi Goto, Taiki Yamaji, Taichi Shimazu, Motoki Iwasaki, Shoichiro Tsugane

**Affiliations:** 1 Epidemiology and Prevention Group, Center for Public Health Sciences, National Cancer Center, Tokyo, Japan; 2 Department of Neuropsychiatry, Keio University School of Medicine, Tokyo, Japan; 3 Precision Health, Department of Bioengineering, Graduate School of Engineering, The University of Tokyo, Tokyo, Japan; 4 Department of Clinical Sciences, Lund University, Skåne University Hospital, Malmö, Sweden; 5 School of Health Innovation, Kanagawa University of Human Services, Kanagawa, Japan; 6 Division of Cancer Statistics Integration, Center for Cancer Control and Information Services, National Cancer Center, Tokyo, Japan; 7 Public Health, Department of Social and Environmental Medicine, Osaka University Graduate School of Medicine, Osaka, Japan; 8 Department of Epidemiology and Prevention, Center for Clinical Sciences, National Center for Global Health and Medicine, Tokyo, Japan

**Keywords:** all-cause mortality, CVD mortality, cancer mortality, general population, Japan, sleep duration

## Abstract

**Background:**

Short and long sleep durations are associated with mortality outcomes. The association between sleep duration and mortality outcomes may differ according to sex and age.

**Methods:**

Participants of the Japan Public Health Center-based prospective study (JPHC Study) were aged 40–69 years and had completed a detailed questionnaire on lifestyle factors. Sex- and age-stratified analyses on the association between habitual sleep duration and mortality from all-causes, cardiovascular diseases (CVD), cancer and other causes included 46,152 men and 53,708 women without a history of CVD or cancer. Cox proportional hazards regression models, adjusted for potential confounders, were used to determine hazard ratios and 95% confidence intervals.

**Results:**

Mean follow-up time was 19.9 years for men and 21.0 years for women. In the multivariable sex-stratified models, some categories of sleep durations ≥8 hours were positively associated with mortality from all-causes, CVD, and other causes in men and women compared with 7 hours. The sex- and age-stratified analyses did not reveal any major differences in the association between sleep duration and mortality outcomes in groups younger and older than 50 years of age. The only exception was the significant interaction between sleep duration and age in women for mortality from other causes.

**Conclusions:**

Sleep durations ≥8 hours are associated with mortality outcomes in men and women. Age may be an effect modifier for the association between sleep duration and mortality from other causes in women.

## INTRODUCTION

Short and long sleep durations are associated with mortality from all-causes,^1^^–^^8^ cardiovascular disease (CVD),^2^^,^^3^ cancer,^2^^,^^9^ and other causes.^10^ These findings have been confirmed also in observational studies from Japan,^11^^–^^19^ a country with the second shortest average sleep duration of all Organisation for Economic Cooperation and Development member states.^20^ Similarly, studies of Japanese populations confirm sex-specific associations between both short^12^^,^^13^^,^^15^^,^^17^ and long^12^^,^^15^^,^^18^ sleep durations and all-cause and cause-specific mortality.

Sex-stratified analyses may, however, be insufficient to explain the mortality risks associated with short and long sleep durations. Older individuals tend to be shorter sleepers,^21^ and older age may influence the association between sleep duration, in particular long sleep duration, and mortality outcomes.^1^ One study found that short sleep duration is associated with all-cause mortality only in men younger than 55 years of age.^22^ Studies investigating age-specific associations are needed. To the best of our knowledge, no study has investigated the age-specific associations between sleep duration and mortality in the Japanese general population.

The aim of the present study was to investigate, using one of the largest Japanese general population cohorts, the sex- and age- (ie, younger or older than 50 years of age) specific associations between sleep duration and mortality from all-causes and major causes (ie, CVD, cancer, and other causes).

## METHODS

The Japan Public Health Center-based prospective Study (JPHC Study) was started in 1990 and conducted in two cohorts, one initiated in 1990 (cohort I) and the other in 1993 (cohort II). The study design has been described in detail elsewhere.^23^ In brief, the baseline population consisted of 140,420 registered Japanese inhabitants. Participants’ age at baseline was 40–59 years for cohort I and 40–69 years for cohort II. All participants were identified by the population registries, which are maintained by the local municipalities in 11 public health center (PHC) areas. Following the exclusion of 303 persons with non-Japanese nationality (*n* = 51), duplicate enrolment (*n* = 10), ineligibility due to an incorrect birth date (*n* = 7), a late report of emigration (*n* = 207), or those who refused further participation (*n* = 28), a population-based cohort of 140,117 individuals was established.

**Figure 1.**
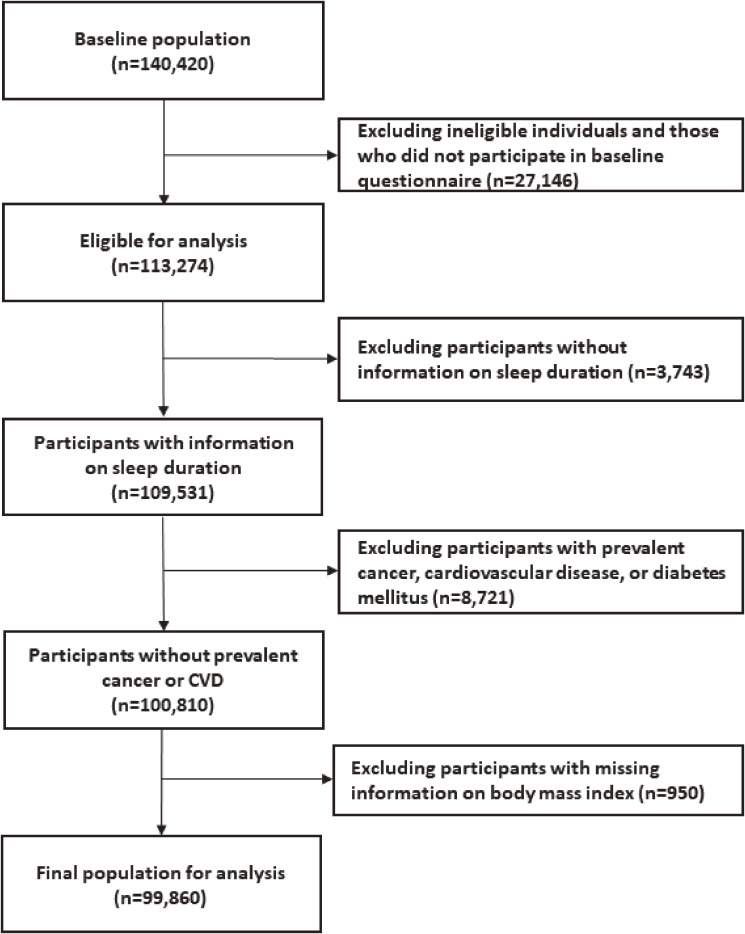
Flowchart of participant inclusion and exclusion

The baseline questionnaires, containing detailed information on medical history and lifestyle, including sleep duration, were returned by 113,274 eligible individuals (81%) (Figure 1). Participants of the present study were excluded if they did not provide information on sleep duration (*n* = 3,743); had a history of cardiovascular disease (*n* = 1,823), cancer (*n* = 2,154) or diabetes mellitus (*n* = 4,744) at baseline; or had missing information on body mass index (BMI) (*n* = 950). A total of 46,152 men and 53,708 women were ultimately included in our analyses.

All persons in the study gave their informed consent prior to their inclusion in the study. The JPHC Study has been approved by the institutional review board of the National Cancer Center, Japan (approval number: 2001-021).

### Follow-up and identification of cause of death

All participants in this study, irrespective of endpoint, were followed from starting point until the end of the follow-up period. The follow-up period ended on December 31, 2014 for all but two PHC areas, Katsushika (December 31, 2009), and Suita (December 31, 2012). Person-years were calculated from starting point to the date of death, emigration from Japan, or end of the follow-up period, whichever came first. For participants who were lost to follow-up (*n* = 819; 0.8%), the censoring date was determined as the last confirmed date of participation in the study.

### Endpoint retrieval

Information on the cause of death for deceased participants was obtained from death certificates, on which the cause of death is defined according to the International Statistical Classification of Diseases and Related Health Problems, tenth revision (ICD-10).^24^ The endpoints of the current study were all-cause mortality, CVD mortality (ICD-10: I20–I25, I60–I69), cancer mortality (ICD-10: C00–C97), and mortality from other causes. Residency registration and death registration are required by the Basic Residential Register Law and Family Registry Law, respectively, and the registers are considered to be complete.

### Sleep duration

Habitual sleep duration was assessed through one question: “How many hours do you usually sleep?”. Answers were provided as integers, which were categorized into six sleep duration groups (≤5, 6, 7, 8, 9, and ≥10 hours). Only 0.5% (*n* = 509) of the study population slept for 4 hours, and only 0.1% (*n* = 82) slept for 11 hours. Subsequently, these groups were combined with the 5 hour and 10 hour groups, respectively. The reference category (7 hours) was chosen on the basis of the lowest all-cause mortality rates in this particular group for both men and women.

### Statistical analyses

Differences in baseline characteristics between sleep duration groups were assessed using the chi-square test or analysis of variance (ANOVA). Sex-specific and age-stratified hazard ratios (HRs) and their 95% confidence intervals (CIs) were determined in Cox proportional hazards models to characterize the relative risk of all-cause mortality, CVD mortality, cancer mortality, and mortality from other causes associated with sleep duration. Age strata were chosen according to the median age (ie, 50 years) of the study population. Median age rather than retirement age was selected as the cut-off given the age distribution of participants in Cohort I (aged 40–59 years) of the JPHC Study.

Model 1 was adjusted for age (continuous), study-area, smoking status (never, former smoker, <20 cigarettes/day, >20 cigarettes/day), alcohol intake (none or occasional, <150, and ≥150 g ethanol/week), green tea consumption (rarely, <1 cup/day, and ≥1 cup/day), and coffee consumption (rarely, <1 cup/day, and ≥1 cup/day). Model 2 for men was adjusted for study area, alcohol intake, living alone (yes/no), health check-up (having undergone any screening examination, such as blood pressure check, blood test, electrocardiography, fundoscopy, chest radiograph, sputum cytology, gastric photofluorography, gastric endoscopy, faecal occult blood test, barium enema, or colonoscopy; yes/no), hypertension (past history of hypertension or use of antihypertensive medication; yes/no) and perceived psychological stress (low, intermediate, or high), and were stratified by age (continuous), BMI (continuous), coffee consumption, physical activity (<1 day/week, ≥1 day/week) and smoking status. Model 2 for women was adjusted for study area, alcohol intake, smoking status, living alone, health check-up, physical activity, hypertension, and stress, and stratified on age, BMI, coffee consumption, and green tea consumption. Indicator terms were created for missing values to allow for their inclusion in the model. The selection of variables for adjustment or stratification in model 2 was conducted on the basis that the model would meet proportional hazards assumptions. Global tests for proportionality were conducted for main analyses and did not reveal any significant deviation from the proportional hazards assumptions. Sensitivity analyses (model 3) excluded the first 5 years of follow-up to minimize the risk of reverse causation.

The *P* value for the interaction between sleep duration and sex and age, respectively, was determined using the likelihood ratio test where model 2 including an interaction term between sleep duration and sex or age was compared to model 2 without this term.

All statistical analyses were performed using Stata version 14.0 SE (StataCorp LP, College Station, TX, USA). Significance levels were two-tailed and set as *α = 0.05*.

## RESULTS

The mean sleep durations of the 46,152 men and 53,708 women included in the study were 7.4 (standard deviation [SD], 1.1) hours and 7.1 (SD, 1.0) hours, respectively. The total follow-up time was 916,419 person-years for men and 1,123,448 person-years for women, corresponding to a mean follow-up time of 19.9 years for men and 21.0 years for women.

Baseline characteristics of the study population stratified by sex according to sleep duration category are shown in Table 1. Overall, there were significant differences between sleep duration categories for all characteristics. Men and women with a sleep duration ≥10 hours were older and were less likely to drink coffee, be physically active, or report high levels of psychological stress. Men with a sleep duration ≤5 hours had the highest BMI, and the highest proportion of high psychological stress, never smokers, alcohol non-consumers, and those living alone. Women with a sleep duration ≤5 hours had the lowest proportion of alcohol non-consumers, health check-up attendees, and the highest proportion of high psychological stress and past/current smokers.

**Table 1.** Baseline characteristics for men and women according to sleep duration

	Sleep duration, hours						*P* value^a^

	<5	6	7	8	9	≥10	
Men (*n* = 46,152)							
Number of individuals	1,612	7,311	15,283	17,509	3,042	1,395	
Proportion of population (%)	3.5	15.8	33.1	37.9	6.6	3.0	
Age, years, mean (SD)	51.1 (8.1)	50.2 (7.8)	50.0 (7.6)	51.6 (8.0)	53.9 (8.2)	55.9 (8.3)	<0.001
Smoking status (%)							<0.001
Never	27.5	24.6	24.1	22.4	22.1	21.3	
Past	23.1	24.0	22.9	23.1	23.8	23.7	
Current <20 cigarettes/day	11.5	12.1	12.8	14.5	18.6	15.8	
Current ≥20 cigarettes/day	37.4	38.9	40.0	39.7	35.1	38.5	
Missing	0.5	0.4	0.2	0.4	0.4	0.7	
Alcohol consumption (%)							<0.001
None/rarely	32.1	29.3	28.8	27.3	25.6	26.0	
<150 g ethanol/week	20.8	23.6	23.9	20.9	17.6	16.4	
≥150 g ethanol/week	41.4	39.3	40.5	45.8	51.3	51.3	
Missing	5.7	7.8	6.9	6.1	5.6	6.3	
Coffee							<0.001
Rarely	27.8	24.1	23.2	30.7	37.3	40.9	
<1 cup/day	27.6	26.2	29.8	30.4	28.6	27.9	
≥1 cup/day	43.5	49.2	46.1	38.0	33.0	30.1	
Missing	1.1	0.6	0.8	0.9	1.2	1.1	
Green tea							<0.001
Rarely	15.0	13.0	10.9	11.4	11.3	12.0	
<1 cup/day	17.4	17.0	16.0	13.5	12.0	12.0	
≥1 cup/day	66.8	69.3	72.5	74.4	75.5	75.0	
Missing	0.8	0.7	0.6	0.7	1.1	1.0	
BMI, kg/m^2^, mean (SD)	24.1 (3.3)	23.8 (3.0)	23.5 (3.0)	23.4 (2.9)	23.1 (2.9)	23.0 (2.9)	<0.001
Physical activity (%)							<0.001
<1 day/week	81.6	79.4	78.9	81.5	82.9	86.6	
≥1 day/week	17.2	20.2	20.5	17.7	16.0	12.9	
Missing	1.2	0.4	0.6	0.9	1.1	0.5	
Living alone (%)	6.5	5.0	3.5	2.7	2.4	3.3	<0.001
Missing	0.2	0.1	0.1	0.1	0.2	0.4	
Health Check-up (%)	80.3	83.0	83.2	82.6	82.8	80.4	0.034
Missing	0.4	0.2	0.2	0.2	0.2	0.1	
Hypertension (%)	24.4	23.3	22.5	24.9	30.5	33.6	<0.001
Missing	0	0	0	0	0	0	
Psychological stress (%)							<0.001
Low	11.1	12.1	11.9	15.4	20.5	20.9	
Intermediate	50.9	55.8	61.6	64.2	63.7	63.1	
High	37.5	31.8	26.1	19.9	15.4	15.1	
Missing	0.5	0.3	0.4	0.5	0.5	0.9	

Women (*n* = 53,708)							
Number of individuals	2,717	11,491	20,627	16,004	2,101	768	
Proportion of population (%)	5.1	21.4	38.4	29.8	3.9	1.4	
Age, years, mean (SD)	51.6 (7.8)	50.2 (7.7)	50.4 (7.7)	52.8 (8.2)	55.7 (8.4)	56.6 (8.6)	<0.001
Smoking status (%)							<0.001
Never	86.3	89.0	90.8	91.1	91.4	88.2	
Past	2.3	2.0	1.9	1.7	1.5	1.4	
Current <20 cigarettes/day	6.4	5.5	4.7	4.6	4.5	5.5	
Current ≥20 cigarettes/day	4.8	3.2	2.3	2.2	2.0	4.0	
Missing	0.2	0.3	0.4	0.4	0.6	0.9	
Alcohol consumption (%)							<0.001
None/rarely	80.8	81.9	83.7	86.6	87.5	88.4	
<150 g ethanol/week	10.7	11.4	10.8	8.6	7.1	4.7	
≥150 g ethanol/week	5.2	2.9	2.1	2.4	3.1	4.7	
Missing	3.3	3.9	3.4	2.5	2.3	2.2	
Coffee							<0.001
Rarely	27.6	24.4	26.9	34.5	43.4	48.7	
<1 cup/day	25.9	27.8	29.5	29.9	28.6	23.1	
≥1 cup/day	45.8	47.1	43.0	34.6	26.7	26.7	
Missing	0.8	0.7	0.6	1.0	1.4	1.6	
Green tea							<0.001
Rarely	16.2	11.8	9.9	11.8	12.2	14.8	
<1 cup/day	14.2	14.2	14.3	12.4	11.0	10.9	
≥1 cup/day	68.4	73.4	75.1	75.0	75.8	72.4	
Missing	1.2	0.7	0.7	0.9	1.1	1.8	
BMI, kg/m^2^, mean (SD)	23.7 (3.5)	23.3 (3.2)	23.2 (3.1)	23.4 (3.2)	23.7 (3.5)	23.9 (3.6)	<0.001
Physical activity (%)							<0.001
<1 day/week	80.1	79.9	79.9	81.5	83.3	85.7	
≥1 day/week	19.0	19.3	19.2	17.2	15.1	12.5	
Missing	0.9	0.8	0.9	1.3	1.6	1.8	
Living alone (%)	6.2	4.4	3.9	5.0	5.5	8.2	<0.001
Missing	0.2	0.1	0.1	0.2	0.3	0.4	
Health Check-up (%)	78.8	80.2	82.3	84.5	87.0	85.3	<0.001
Missing	0.3	0.3	0.3	0.3	0.3	0.1	
Hypertension (%)	20.0	18.9	19.7	24.1	32.5	31.8	<0.001
Missing	0	0	0	0	0	0	
Psychological stress (%)							<0.001
Low	13.2	13.9	16.3	18.3	22.8	22.1	
Intermediate	55.8	61.1	64.6	66.9	64.0	62.6	
High	30.7	24.6	18.7	14.2	12.4	14.3	
Missing	0.4	0.4	0.4	0.7	0.8	0.9	

BMI, body mass index; SD, standard deviation.

^a^χ^2^ test was used for categorical variables and ANOVA for continuous variables.

### All-cause mortality

There were a total of 11,259 deaths among men and 6,783 deaths among women during follow-up. In the multivariable analysis, and when compared to a sleep duration of 7 hours, men had a significantly increased risk of all-cause mortality with a sleep duration of ≥10 hours (HR 1.83; 95% CI, 1.33–2.52) (Table 2). Women had a significantly increased risk of all-cause mortality with sleep durations of 8 hours (HR 1.22; 95% CI, 1.08–1.37), and ≥10 hours (HR 1.72; 95% CI, 1.22–2.43), respectively.

**Table 2.** Hazard ratios and their confidence intervals for mortality according to sleep duration for men and women

	Men (*n* = 46,152)						Women (*n* = 53,708)					
	
	Sleep duration, hours						Sleep duration, hours					
	
	≤5	6	7	8	9	≥10	≤5	6	7	8	9	≥10
Person-years	31,015	143,767	307,633	349,982	58,717	25,306	56,157	238,019	434,390	336,613	43,282	14,988

**Cause of death**												
**All-cause**												
Number (Events)	1,612 (429)	7,311 (1,544)	15,283 (3,054)	17,509 (4,569)	3,042 (1,021)	1,395 (642)	2,717 (354)	11,491 (1,175)	20,627 (2,124)	16,004 (2,448)	2,101 (458)	768 (224)
Model 1^a^, HR (95% CI)	1.31^***^ (1.18, 1.45)	1.08^*^ (1.01, 1.14)	Reference	1.12^***^ (1.06, 1.17)	1.23^***^ (1.14, 1.32)	1.54^***^ (1.41, 1.67)	1.11 (0.99, 1.24)	1.03 (0.96, 1.10)	Reference	1.16^***^ (1.10, 1.23)	1.30^***^ (1.17, 1.44)	1.67^***^ (1.45, 1.92)
Model 2^b^, HR (95% CI)	1.28 (0.89, 1.85)	1.05 (0.86, 1.28)	Reference	1.11 (0.95, 1.29)	1.17 (0.92, 1.49)	1.83^***^ (1.33, 2.52)	0.99 (0.77, 1.27)	0.96 (0.83, 1.11)	Reference	1.22^***^ (1.08, 1.37)	1.23 (0.98, 1.56)	1.72^**^ (1.22, 2.43)
Model 3^c^, HR (95% CI)	1.25 (0.85, 1.85)	0.99 (0.79, 1.23)	Reference	1.06 (0.91, 1.25)	1.09 (0.84, 1.40)	1.60^**^ (1.14, 2.25)	0.91 (0.70, 1.19)	0.95 (0.81, 1.10)	Reference	1.21^**^ (1.06, 1.37)	1.19 (0.93, 1.52)	1.60^*^ (1.09, 2.34)

**CVD**												
Number (Events)	1,612 (72)	7,311 (241)	15,283 (451)	17,509 (667)	3,042 (148)	1,395 (112)	2,717 (66)	11,491 (176)	20,627 (291)	16,004 (395)	2,101 (92)	768 (34)
Model 1^a^, HR (95% CI)	1.48^**^ (1.16, 1.90)	1.14 (0.97, 1.33)	Reference	1.09 (0.97, 1.23)	1.20 (0.99, 1.45)	1.80^***^ (1.46, 2.22)	1.43^*^ (1.09, 1.86)	1.12 (0.93, 1.35)	Reference	1.31^***^ (1.12, 1.52)	1.74^***^ (1.37, 2.21)	1.65^**^ (1.15, 2.36)
Model 2^b^, HR (95% CI)	1.36 (0.45, 4.08)	1.42 (0.77, 2.61)	Reference	1.39 (0.91, 2.12)	2.04^*^ (1.03, 4.02)	3.61^**^ (1.46, 8.94)	0.71 (0.36, 1.40)	0.81 (0.54, 1.23)	Reference	1.20 (0.86, 1.67)	1.61 (0.90, 2.90)	2.71 (1.00, 7.37)
Model 3^c^, HR (95% CI)	1.46 (0.46, 4.62)	1.54 (0.80, 3.00)	Reference	1.14 (0.72, 1.81)	1.45 (0.69, 3.02)	2.39 (0.91, 6.25)	0.83 (0.41, 1.66)	0.91 (0.59, 1.41)	Reference	1.31 (0.92, 1.86)	1.74 (0.95, 3.19)	2.11 (0.71, 6.23)

**Cancer**												
Number (Events)	1,612 (161)	7,311 (629)	15,283 (1,270)	17,509 (1,874)	3,042 (407)	1,395 (205)	2,717 (134)	11,491 (482)	20,627 (889)	16,004 (906)	2,101 (115)	768 (71)
Model 1^a^, HR (95% CI)	1.20^*^ (1.02, 1.42)	1.06 (0.97, 1.17)	Reference	1.10^**^ (1.03, 1.19)	1.19^**^ (1.06, 1.33)	1.18^*^ (1.02, 1.37)	1.05 (0.88, 1.26)	1.00 (0.90, 1.12)	Reference	1.11^*^ (1.01, 1.21)	0.90 (0.74, 1.10)	1.50^***^ (1.18, 1.92)
Model 2^b^, HR (95% CI)	1.56 (0.89, 2.74)	0.95 (0.70, 1.27)	Reference	1.04 (0.83, 1.31)	1.16 (0.82, 1.66)	1.05 (0.62, 1.78)	0.95 (0.66, 1.38)	1.08 (0.88, 1.34)	Reference	1.15 (0.96, 1.38)	0.89 (0.59, 1.34)	1.53 (0.87, 2.70)
Model 3^c^, HR (95% CI)	1.32 (0.71, 2.47)	0.83 (0.61, 1.14)	Reference	0.97 (0.76, 1.25)	1.14 (0.78, 1.66)	0.91 (0.52, 1.58)	0.91 (0.62, 1.35)	0.99 (0.79, 1.24)	Reference	1.10 (0.91, 1.34)	0.85 (0.55, 1.31)	1.58 (0.85, 2.94)

**Other cause**												
Number (Events)	1,612 (196)	7,311 (674)	15,283 (1,333)	17,509 (2,028)	3,042 (466)	1,395 (325)	2,717 (154)	11,491 (517)	20,627 (944)	16,004 (1,147)	2,101 (251)	768 (119)
Model 1^a^, HR (95% CI)	1.35^***^ (1.16, 1.57)	1.07 (0.97, 1.17)	Reference	1.13^***^ (1.06, 1.21)	1.27^***^ (1.14, 1.41)	1.78^***^ (1.58, 2.01)	1.06 (0.89, 1.26)	1.02 (0.92, 1.14)	Reference	1.17^***^ (1.08, 1.28)	1.47^***^ (1.27, 1.69)	1.80^***^ (1.48, 2.18)
Model 2^b^, HR (95% CI)	1.08 (0.62, 1.87)	1.11 (0.79, 1.54)	Reference	1.09 (0.87, 1.37)	0.97 (0.66, 1.41)	2.32^*^ (1.44, 3.74)	1.14 (0.76, 1.72)	0.89 (0.71, 1.23)	Reference	1.27^**^ (1.05, 1.53)	1.40 (0.99, 1.98)	1.75^*^ (1.07, 2.88)
Model 3^c^, HR (95% CI)	1.17 (0.66, 2.09)	1.09 (0.76, 1.55)	Reference	1.13 (0.89, 1.44)	0.90 (0.60, 1.35)	2.27^**^ (1.36, 3.80)	0.93 (0.60, 1.45)	0.91 (0.72, 1.16)	Reference	1.25^*^ (1.02, 1.52)	1.30 (0.90, 1.86)	1.64 (0.95, 2.84)

CI, confidence interval; CVD, cardiovascular disease; HR, hazard ratio.

^a^Model 1(men and women) was adjusted for age, study area, smoking, alcohol intake, green tea consumption, and coffee consumption.

^b^Model 2(men) adjusted for study area, alcohol intake, green tea consumption, living alone, health check-up, history of hypertension, and stress, and stratified on age, body mass index, coffee consumption, physical activity, and smoking.

^b^Model 2(women) adjusted for study area, alcohol intake, smoking, living alone, health check-up, physical activity, history of hypertension, and stress, and stratified on age, body mass index, coffee consumption, and green tea consumption.

^c^Model 3 adjusts and stratifies as model 2 and further excludes those who died in the first 5 years of analysis.

Bold values denote statistically significant result; ^*^P < 0.05, ^**^P < 0.01, ^***^P < 0.001 vs reference.

Table 3 and Table 4 show the sex- and age-stratified analyses. The interaction between age and sleep duration for all-cause mortality was not significant for either men or women. Sensitivity analyses which excluded the first 5 years of follow-up did not markedly change the found associations.

**Table 3.** Hazard ratios and their confidence intervals for mortality according to sleep duration in men stratified by age (younger/older than 50 years of age)

	Age ≤50 (*n* = 24,188)						Age >50 (*n* = 21,964)					
	
	Sleep duration, hours						Sleep duration, hours					
	
	≤5	6	7	8	9	≥10	≤5	6	7	8	9	≥10
Person-years	16,884	83,641	183,373	181,280	23,989	8,305	14,130	60,126	124,260	168,702	34,728	17,001

**Cause of death**												
**All-cause**												
Number (Events)	844 (119)	4,173 (416)	8,952 (940)	8,663 (1,056)	1,146 (192)	410 (94)	768 (310)	3,138 (1,128)	6,331 (2,114)	8,846 (3,513)	1,896 (829)	985 (548)
Model 1^a^, HR (95% CI)	1.37^***^ (1.13, 1.66)	1.00 (0.89, 1.12)	Reference	1.08 (0.99, 1.18)	1.41^***^ (1.21, 1.65)	1.95^***^ (1.58, 2.41)	1.29^***^ (1.14, 1.45)	1.09^*^ (1.01, 1.17)	Reference	1.20^***^ (1.13, 1.26)	1.33^***^ (1.23, 1.44)	1.79^***^ (1.63, 1.97)
Model 2^b^, HR (95% CI)	1.53^***^ (1.18, 1.98)	1.01 (0.86, 1.18)	Reference	1.13 (1.00, 1.27)	1.30^*^ (1.05, 1.62)	2.07^***^ (1.52, 2.81)	1.47^***^ (1.23, 1.77)	1.08 (0.97, 1.20)	Reference	1.16^***^ (1.07, 1.25)	1.28^***^ (1.13, 1.44)	1.61^***^ (1.39, 1.85)
Model 3^c^, HR (95% CI)	1.59^***^ (1.21, 2.09)	1.00 (0.85, 1.18)	Reference	1.11 (0.98, 1.26)	1.26^*^ (1.00, 1.59)	1.95^***^ (1.41, 2.72)	1.41^***^ (1.15, 1.72)	1.08 (0.96, 1.21)	Reference	1.17^***^ (1.07, 1.27)	1.21^**^ (1.06, 1.38)	1.61^***^ (1.38, 1.87)

**CVD**												
Number (Events)	844 (21)	4,173 (64)	8,952 (130)	8,663 (158)	1,146 (30)	410 (12)	768 (51)	3,138 (177)	6,331 (321)	8,846 (509)	1,896 (118)	985 (100)
Model 1^a^, HR (95% CI)	1.76^*^ (1.11, 2.80)	1.12 (0.83, 1.51)	Reference	1.15 (0.91, 1.46)	1.57^*^ (1.06, 2.35)	1.78 (0.98, 3.22)	1.39^*^ (1.03, 1.87)	1.13 (0.94, 1.35)	Reference	1.14 (0.99, 1.31)	1.26^*^ (1.02, 1.56)	2.19^***^ (1.74, 2.74)
Model 2^b^, HR (95% CI)	1.73 (0.94, 3.21)	0.86 (0.56, 1.30)	Reference	0.94 (0.68, 1.30)	1.22 (0.70, 2.14)	1.84 (0.80, 4.23)	1.85^**^ (1.18, 2.90)	1.22 (0.93, 1.61)	Reference	1.05 (0.86, 1.29)	1.12 (0.82, 1.54)	2.05^***^ (1.44, 2.92)
Model 3^c^, HR (95% CI)	1.65 (0.85, 3.19)	0.87 (0.56, 1.34)	Reference	0.94 (0.67, 1.33)	1.17 (0.64, 2.13)	1.68 (0.70, 4.03)	1.66^*^ (1.02, 2.69)	1.28 (0.96, 1.72)	Reference	1.04 (0.84, 1.29)	1.06 (0.75, 1.49)	2.04^***^ (1.39, 2.99)

**Cancer**												
Number (Events)	844 (36)	4,173 (168)	8,952 (395)	8,663 (412)	1,146 (65)	410 (33)	768 (125)	3,138 (461)	6,331 (875)	8,846 (1,462)	1,896 (342)	985 (172)
Model 1^a^, HR (95% CI)	1.01 (0.72, 1.43)	0.97 (0.81, 1.16)	Reference	1.00 (0.87, 1.14)	1.14 (0.88, 1.48)	1.65^**^ (1.15, 2.35)	1.28^*^ (1.06, 1.54)	1.08 (0.97, 1.21)	Reference	1.20^***^ (1.10, 1.31)	1.33^***^ (1.18, 1.51)	1.35^***^ (1.14, 1.59)
Model 2^b^, HR (95% CI)	1.24 (0.79, 1.94)	1.00 (0.78, 1.27)	Reference	1.04 (0.86, 1.25)	1.06 (0.74, 1.52)	1.84^*^ (1.13, 3.00)	1.59^**^ (1.19, 2.13)	1.06 (0.90, 1.25)	Reference	1.19^**^ (1.06, 1.35)	1.38^***^ (1.15, 1.66)	1.30^*^ (1.02, 1.64)
Model 3^c^, HR (95% CI)	1.32 (0.83, 2.10)	1.00 (0.78, 1.29)	Reference	1.06 (0.87, 1.28)	1.08 (0.74, 1.56)	1.75^*^ (1.04, 2.96)	1.49^*^ (1.08, 2.07)	1.09 (0.92, 1.30)	Reference	1.23^**^ (1.08, 1.40)	1.30^*^ (1.06, 1.60)	1.36^*^ (1.05, 1.76)

**Other cause**												
Number (Events)	844 (62)	4,173 (184)	8,952 (415)	8,663 (486)	1,146 (97)	410 (49)	768 (134)	3,138 (490)	6,331 (918)	8,846 (1,542)	1,896 (369)	985 (276)
Model 1^a^, HR (95% CI)	1.58^***^ (1.21, 2.07)	0.99 (0.84, 1.18)	Reference	1.13 (0.99, 1.29)	1.61^***^ (1.29, 2.02)	2.29^***^ (1.70, 3.08)	1.27^**^ (1.06, 1.52)	1.08 (0.97, 1.21)	Reference	1.21^***^ (1.11, 1.31)	1.35^***^ (1.20, 1.52)	2.09^***^ (1.83, 2.40)
Model 2^b^, HR (95% CI)	1.73^**^ (1.18, 2.53)	1.08 (0.85, 1.37)	Reference	1.29^**^ (1.07, 1.55)	1.52^*^ (1.10, 2.10)	2.39^***^ (1.52, 3.76)	1.24 (0.93, 1.65)	1.06 (0.90, 1.25)	Reference	1.15^*^ (1.02, 1.30)	1.23^*^ (1.03, 1.48)	1.71^***^ (1.39, 2.12)
Model 3^c^, HR (95% CI)	1.83^*^ (1.23, 2.73)	1.05 (0.81, 1.36)	Reference	1.23^*^ (1.02, 1.50)	1.45^*^ (1.03, 2.05)	2.25^***^ (1.38, 3.66)	1.26 (0.93, 1.71)	1.01 (0.84, 1.20)	Reference	1.14^*^ (1.00, 1.30)	1.18 (0.97, 1.43)	1.63^***^ (1.30, 2.04)

CI, confidence interval; CVD, cardiovascular disease; HR, hazard ratio.

^a^Model 1 was adjusted for study area, smoking, alcohol intake, green tea consumption, and coffee consumption.

^b^Model 2 was adjusted for study area, alcohol intake, green tea consumption, living alone, health check-up, history of hypertension, and stress, and stratified on body mass index, coffee consumption, physical activity, and smoking.

^c^Model 3 adjusts and stratifies as model 2 and further excludes those who died in the first 5 years of analysis.

Bold values denote statistically significant result; ^*^P < 0.05, ^**^P < 0.01, ^***^P < 0.001 vs reference.

**Table 4.** Hazard ratios and their confidence intervals for mortality according to sleep duration in women stratified by age (younger/older than 50 years of age)

	Age ≤50 (*n* = 27,586)						Age >50 (*n* = 26,122)					
	
	Sleep duration, hours						Sleep duration, hours					
	
	≤5	6	7	8	9	≥10	≤5	6	7	8	9	≥10
Person-years	29,344	140,228	245,824	146,762	13,294	4,024	26,813	97,791	188,566	189,851	29,988	10,964

**Cause of death**												
**All-cause**												
Number (Events)	1,416 (81)	6,803 (335)	11,704 (555)	6,852 (454)	615 (59)	196 (28)	1,301 (273)	4,688 (840)	8,923 (1,569)	9,152 (1,994)	1,486 (399)	572 (196)
Model 1^a^, HR (95% CI)	1.18 (0.94, 1.50)	1.06 (0.93, 1.22)	Reference	1.31^***^ (1.16, 1.49)	1.80^***^ (1.38, 2.36)	2.68^***^ (1.83, 3.92)	1.16^*^ (1.02, 1.32)	1.02 (0.94, 1.11)	Reference	1.22^***^ (1.14, 1.30)	1.48^***^ (1.32, 1.65)	1.95^***^ (1.68, 2.26)
Model 2^b^, HR (95% CI)	1.24 (0.93, 1.65)	1.05 (0.90, 1.23)	Reference	1.34^***^ (1.15, 1.55)	1.75^***^ (1.24, 2.48)	2.03^**^ (1.21, 3.40)	1.13 (0.97, 1.32)	1.00 (0.91, 1.10)	Reference	1.20^***^ (1.11, 1.30)	1.38^***^ (1.21, 1.57)	1.81^***^ (1.51, 2.17)
Model 3^c^, HR (95% CI)	1.24 (0.91, 1.67)	1.08 (0.91, 1.28)	Reference	1.33^***^ (1.14, 1.56)	1.77^**^ (1.23, 2.54)	2.29^**^ (1.31, 4.02)	1.10 (0.93, 1.30)	1.00 (0.90, 1.10)	Reference	1.20^***^ (1.11, 1.30)	1.35^***^ (1.18, 1.55)	1.66^***^ (1.36, 2.02)

**CVD**												
Number (Events)	1,416 (9)	6,803 (42)	11,704 (67)	6,852 (59)	615 (10)	196 (6)	1,301 (57)	4,688 (134)	8,923 (224)	9,152 (336)	1,486 (82)	572 (28)
Model 1^a^, HR (95% CI)	1.01 (0.50, 2.03)	1.08 (0.74, 1.60)	Reference	1.34 (0.94, 1.90)	2.29^*^ (1.18, 4.47)	3.97^***^ (1.70, 9.24)	1.66^***^ (1.24, 2.22)	1.13 (0.91, 1.40)	Reference	1.42^***^ (1.20, 1.68)	2.09^***^ (1.62, 2.69)	1.89^**^ (1.27, 2.80)
Model 2^b^, HR (95% CI)	0.70 (0.28, 1.75)	0.92 (0.56, 1.51)	Reference	1.35 (0.86, 2.11)	1.99 (0.73, 5.46)	3.72 (0.96, 14.48)	1.42 (0.96, 2.08)	1.10 (0.85, 1.42)	Reference	1.36^**^ (1.11, 1.67)	2.16^***^ (1.60, 2.93)	1.22 (0.73, 2.03)
Model 3^c^, HR (95% CI)	0.59 (0.22, 1.62)	1.03 (0.59, 1.77)	Reference	1.56 (0.96, 2.53)	1.95 (0.66, 5.71)	3.66 (0.68, 19.63)	1.47 (0.99, 2.19)	1.13 (0.87, 1.47)	Reference	1.37^**^ (1.11, 1.69)	2.11^***^ (1.54, 2.90)	0.94 (0.53, 1.68)

**Cancer**												
Number (Events)	1,416 (41)	6,803 (190)	11,704 (298)	6,852 (226)	615 (16)	196 (5)	1,301 (93)	4,688 (292)	8,923 (591)	9,152 (680)	1,486 (99)	572 (66)
Model 1^a^, HR (95% CI)	1.13 (0.82, 1.57)	1.12 (0.94, 1.35)	Reference	1.24^*^ (1.04, 1.48)	0.96 (0.58, 1.58)	0.95 (0.39, 2.30)	1.07 (0.86, 1.34)	0.95 (0.82, 1.09)	Reference	1.12^*^ (1.00, 1.25)	1.01 (0.81, 1.25)	1.82^***^ (1.41, 2.36)
Model 2^b^, HR (95% CI)	1.31 (0.90, 1.92)	1.15 (0.93, 1.41)	Reference	1.24^*^ (1.02, 1.52)	0.94 (0.52, 1.70)	0.60 (0.18, 2.00)	1.06 (0.81, 1.37)	0.99 (0.85, 1.17)	Reference	1.16^*^ (1.02, 1.32)	0.99 (0.77, 1.26)	1.80^***^ (1.32, 2.44)
Model 3^c^, HR (95% CI)	1.33 (0.89, 1.98)	1.10 (0.88, 1.37)	Reference	1.24^*^ (1.00, 1.53)	0.95 (0.51, 1.75)	0.68 (0.20, 2.31)	1.03 (0.78, 1.37)	0.93 (0.79, 1.11)	Reference	1.15^*^ (1.00, 1.32)	0.98 (0.75, 1.27)	1.68^**^ (1.21, 2.35)

**Other cause**												
Number (Events)	1,416 (31)	6,803 (103)	11,704 (190)	6,852 (169)	615 (33)	196 (17)	1,301 (123)	4,688 (414)	8,923 (754)	9,152 (978)	1,486 (218)	572 (102)
Model 1^a^, HR (95% CI)	1.33 (0.91, 1.95)	0.96 (0.76, 1.22)	Reference	1.41^***^ (1.15, 1.74)	2.86^***^ (1.97, 4.15)	4.65^***^ (2.81, 7.67)	1.09 (0.90, 1.32)	1.04 (0.93, 1.18)	Reference	1.23^***^ (1.12, 1.36)	1.65^***^ (1.42, 1.92)	2.07^***^ (1.68, 2.55)
Model 2^b^, HR (95% CI)	1.38 (0.85, 2.26)	0.95 (0.71, 1.28)	Reference	1.50^**^ (1.16, 1.95)	3.05^***^ (1.83, 5.10)	3.74^***^ (1.81, 7.71)	1.10 (0.87, 1.38)	0.98 (0.85, 1.13)	Reference	1.18^**^ (1.06, 1.33)	1.45^***^ (1.21, 1.75)	2.07^***^ (1.60, 2.68)
Model 3^c^, HR (95% CI)	1.42 (0.83, 2.40)	1.09 (0.81, 1.48)	Reference	1.42^*^ (1.08, 1.88)	3.09^***^ (1.80, 5.32)	4.91^***^ (2.23, 10.83)	1.03 (0.81, 1.31)	1.01 (0.87, 1.16)	Reference	1.19^**^ (1.06, 1.33)	1.40^***^ (1.15, 1.71)	1.94^***^ (1.47, 2.55)

CI, confidence interval; CVD, cardiovascular disease; HR, hazard ratio.

^a^Model 1 was adjusted for study area, smoking, alcohol intake, green tea consumption, and coffee consumption.

^b^Model 2 was adjusted for study area, alcohol intake, smoking, living alone, health check-up, physical activity, history of hypertension, and stress, and stratified on body mass index, green tea consumption, and coffee consumption.

^c^Model 3 adjusts and stratifies as model 2 and further excludes those who died in the first 5 years of analysis.

Bold values denote statistically significant result; ^*^P < 0.05, ^**^P < 0.01, ^***^P < 0.001 vs reference.

### CVD-mortality

The multivariable adjusted models showed an increased risk of CVD mortality for men with sleep durations of 9 hours (HR 2.04; 95% CI, 1.03–4.02), and ≥10 hours (HR 3.61; 95% CI, 1.46–8.94). For women, there was no increased risk of CVD mortality with any sleep duration compared to 7 hours. Sensitivity analyses excluding the first 5 years of follow-up abrogated the results for men.

The interaction between age and sleep duration for CVD mortality was not significant in either men or women. Sensitivity analyses excluding the first 5 years of follow-up did not result in any marked changes of the found associations.

### Cancer mortality

Sleep duration was not associated with cancer mortality in men or women when stratified only by sex. The interaction between age and sleep duration for cancer mortality was not significant in either men or women. Sensitivity analyses excluding the first 5 years of follow-up did not result in any marked changes of the found associations.

### Other causes of death

Men had an increased risk of other causes of death with a sleep duration of ≥10 hours (HR 2.32; 95% CI, 1.44–3.74). For women, the risk of death from other causes was increased among those with sleep durations of 8 hours (HR 1.27; 95% CI, 1.05–1.53), and 10 hours (HR 1.75; 95% CI, 1.07–2.88). Sensitivity analyses excluding the first 5 years of follow-up abrogated the significant association for women with a sleep duration of 10 hours.

The interaction between age and sleep duration for mortality from other causes was significant for women (*P* = 0.023) but not for men. In the sex- and age-stratified analyses, women <50 years of age had an increased risk of mortality from other causes with sleep durations of 8 hours (HR 1.50; 95% CI, 1.16–1.95), 9 hours (HR 3.05; 95% CI, 1.83–5.10), and ≥10 hours (HR 3.74; 95% CI, 1.81–7.71). Women ≥50 years of age had an increased risk of mortality from other causes with sleep durations of 8 hours (HR 1.18; 95% CI, 1.06–1.33), 9 hours (HR 1.45; 95% CI, 1.21–1.75), and ≥10 hours (HR 2.07; 95% CI, 1.60–2.68). Sensitivity analyses excluding the first 5 years of follow-up did not result in any marked changes of the found associations.

## DISCUSSION

The findings of this study show that, overall, sleep durations ≥8 hours are associated with mortality from all-cause, CVD, and other causes in the Japanese general population. The sex- and age-stratified results further indicate that age, with a cut-off at 50 years, may act as an effect modifier for the association between sleep duration and the risk of other-cause mortality in women.

Our findings of an increased risk of mortality with sleep durations ≥8 hours in men and women is supported by a number of previous studies which in Japanese populations have found increased sex-specific risks with only long sleep duration for all-cause mortality in men^14^^,^^15^^,^^18^ and women,^12^^,^^14^^,^^18^ as well as for CVD in men^11^^,^^18^ and women.^11^^,^^12^^,^^14^ Our sex-stratified analyses indicate similar mortality risks between men and women for all outcomes. This is highlighted by the non-significant interaction between sex and sleep duration for each of the respective mortality outcomes. Contrary to the results for sex, however, age was an effect modifier for the association between sleep duration and mortality from other causes among women. In the present study, the mortality from other causes endpoint was composed of wide-ranging causes of death, including infections, inflammations, accidents, injuries, and suicide. Any speculation as to the possible cause of age as an effect modifier is, therefore, inherently difficult. Additionally, given our stratification and the resulting low number of cases, it would not be possible to conduct cause-specific analyses due to the risk of losing statistical power. However, our sensitivity analyses, which excluded the first 5 years of follow-up, indicate that the found associations are unlikely to be due to reverse causation. Future studies are, therefore, encouraged to investigate this further.

This study did not find any association between sleep duration and cancer mortality in the sex-stratified analyses, which is in accordance with a large number of studies that have found no association between sleep duration and cancer mortality.^11^^,^^19^^,^^25^^–^^29^ However, two meta-analyses have shown that longer, not shorter sleep durations, are associated with increased risk of cancer mortality.^2^^,^^9^ Indeed, when further stratifying our analyses by age, an increased risk of cancer mortality in women was found with sleep durations of 8 hours in both younger and older women and with 10 hours of sleep in older women. Among men, those younger than 50 years of age were at an increased risk of cancer mortality with sleep durations ≥10 hours, whereas men older than 50 years of age were at an increased risk with all sleep durations except for 6 hours. One possible explanation for the finding in younger and older women could be the increased risk of breast cancer with increasing sleep duration^30^; our findings could indicate the possibility of an increased risk of mortality from site-specific cancers, such as breast cancer, in women with sleep durations ≥8 hours. It was only in older men that sleep durations shorter than 7 hours were associated with cancer mortality. This is in accordance with one Japanese study, which found that the risk of cancer mortality was increased with sleep durations shorter than 6 hours only in men.^12^ However, that study did not further stratify results by age, making it difficult to draw any further parallels with our own results. Overall, older age is a strong risk factor for cancer mortality, which could serve as an explanation for our findings in men older than 50 years of age. Future studies should attempt to investigate the association between sleep duration and site-specific cancer mortality in both younger and older men and women. However, the large population size and long follow-up time of the present study would be considered prerequisites to identify associations in two strata.

It is notable that, in the multivariable adjusted sex-stratified analyses in our study, sleep durations shorter than 7 hours were not associated with any increased risk of mortality in either men or women. This result contrasts a large number of studies in which short sleep duration is associated with mortality from all-causes,^1^^–^^5^^,^^8^^,^^10^^–^^13^^,^^15^^–^^17^^,^^25^^–^^27^^,^^29^^,^^31^^–^^34^ CVD,^10^^,^^25^^–^^27^^,^^29^ cancer,^10^^,^^12^ and other causes.^10^^–^^12^^,^^27^ Among the men in our study, it is clear that the non-association between sleep durations <7 hours and mortality is explained by the adjusting and stratifying covariates, which abrogate the significant associations found in all of the minimally adjusted models. Furthermore, based on the age-stratified models and interactions between age and sleep duration, it is also clear that age with a cut-off at 50 years is not an effect modifier in the present study for the associations between sleep duration and mortality from all-causes, CVD, and cancer. This is in contrast to a study that found short sleep duration associated with all-cause mortality only in men younger than 55 years of age.^22^ Although the reason for the discrepancy between our own result and previous studies is difficult to ascertain, they may very well be due to methodological differences, such as population size, follow-up time, and adjusting covariates.

Our found associations between sleep duration and mortality outcomes are similar in both men and women irrespective of age group. The only exception is the significant interaction between age and sleep duration for mortality from other causes among women. On closer inspection, results indicate significantly larger effect sizes for sleep durations ≥9 hours in women who are younger than 50 years of age. A precise assessment of the reason for this discrepancy is complicated by the heterogenous causes of death included in the mortality from other causes endpoint. Additionally, it is not inconceivable that results could be different with the selection of a higher age cut-off for stratification. Indeed, a recent study found that the risk of mortality with short and long sleep durations was increased only in individuals younger than 65 years of age.^28^ Similarly, a meta-analysis reported of an association between short and long sleep durations and all-cause mortality only in individuals older than 60 years of age.^31^ The present study could not set a different age cut-off due to the age distribution of the study participants in the two cohorts. Future studies with a sufficient number of participants are advised to select higher age cut-offs to investigate the associations between sleep duration and mortality outcomes in sex-stratified analyses.

The biological mechanisms for the association between short and long sleep durations and mortality are yet to be established and are beyond the scope of the current study. Short sleep duration could potentially be causally related to adverse health outcomes due to its association with decreased leptin and increased ghrelin,^35^ which could result in increased appetite and lead to increased BMI. High BMI, in turn, is associated with all-cause-, CVD-, cancer-, and other-cause mortality in East Asian populations.^36^ Moreover, the increased risk of mortality with sleep duration could be associated with obstructive sleep apnea. Conversely, the association between long sleep duration and mortality could be due to comorbidity^37^ or residual confounding.^38^

This study has a few limitations. Sleep duration was self-reported. Although self-reported sleep duration may overestimate actual sleep time,^39^ this would result in non-differential misclassification and an underestimation of our study findings. Second, we were unable to adjust for potentially important confounders, such as socioeconomic status, education, shift work, depression, sleep apnea, or sleep quality. Third, the present study asked about sleep duration in general and did not distinguish between daytime and night-time sleep duration. Results can, therefore, not be considered in the context of daytime napping. Fourth, our findings may not be generalizable to other populations. Fifth, the selected age cut-off of 50 years was based on the median value as we were unable to, due to the age distribution of the population, select a higher cut-off (eg, based on retirement age). Finally, the observational nature of the study makes it difficult to prove causality.

Despite these limitations, this study also has a number of strengths. First, the study population is highly representative of the Japanese general population, with good generalizability of results. Second, it is one of the largest studies to date to investigate the interaction between sleep duration and sex and age, respectively, and allow for the stratification of results. Third, we have adjusted for a large number of known and important confounders for the association between sleep duration and mortality. Fourth, we have excluded individuals with cancer, cardiovascular disease, and diabetes mellitus at baseline, and conducted sensitivity analyses excluding the first 5 years of follow-up, thereby minimizing the risk of reverse causation bias.

### Conclusion

This study has found that only sleep durations ≥8 hours are associated with mortality outcomes in men and women. Additionally, age may be an effect modifier for the association between sleep duration and mortality from other causes in women.
